# Poorer outcomes among cancer patients diagnosed with *Clostridium difficile* infections in United States community hospitals

**DOI:** 10.1186/s12879-017-2553-z

**Published:** 2017-06-23

**Authors:** Andrew Delgado, Ivan A. Reveles, Felicia T. Cabello, Kelly R. Reveles

**Affiliations:** 1 College of Pharmacy, The University of Texas at Austin, 2409 University Avenue, A1900, Austin, TX 78712 USA; 2 Pharmacotherapy Education and Research Center, The University of Texas Health Science Center at San Antonio, 7703 Floyd Curl Drive, MC-6220, San Antonio, TX 78229 USA; 3 Cancer Therapy and Research Center, The University of Texas Health Science Center at San Antonio, 7979 Wurzbach Road, San Antonio, TX 78229 USA

**Keywords:** *Clostridium Difficile*, Cancer, Epidemiology, Mortality

## Abstract

**Background:**

Cancer predisposes patients to *Clostridium difficile* infection (CDI) due to health care exposures and medications that disrupt the gut microbiota or reduce immune response. Despite this association, the national rate of CDI among cancer patients is unknown. Furthermore, it is unclear how CDI affects clinical outcomes in cancer. The objective of this study was to describe CDI incidence and health outcomes nationally among cancer patients in the United States (U.S.).

**Methods:**

Data for this study were obtained from the U.S. National Hospital Discharge Surveys from 2001 to 2010. Eligible patients included those at least 18 years old with a discharge diagnosis of cancer (ICD-9-CM codes 140–165.X, 170–176.X, 179–189.X, 190–209.XX). CDI was identified using ICD-9-CM code 008.45. Data weights were applied to sampled patients to provide national estimates. CDI incidence was calculated as CDI discharges per 1000 total cancer discharges. The in-hospital mortality rate and hospital length of stay (LOS) were compared between cancer patients with and without CDI using bivariable analyses.

**Results:**

A total of 30,244,426 cancer discharges were included for analysis. The overall incidence of CDI was 8.6 per 1000 cancer discharges. CDI incidence increased over the study period, peaking in 2008 (17.2 per 1000 cancer discharges). Compared to patients without CDI, patients with CDI had significantly higher mortality (9.4% vs. 7.5%, *p* < 0.0001) and longer median LOS (9 days vs. 4 days, *p* < 0.0001).

**Conclusions:**

CDI incidence is increasing nationally among cancer patients admitted to U.S. community hospitals. CDI was associated with significantly increased mortality and hospital LOS.

## Background

*Clostridium difficile* is the most common pathogen contributing to healthcare-associated infections [1]. This Gram-positive, anaerobic bacterium can colonize the human gut, typically following health care contact and exposure to agents that disrupt the normal gut microbiota, like antibiotics. Patients may then develop a toxin-mediated intestinal disease, *Clostridium difficile* infection (CDI). CDI results in frequent diarrhea, but may also progress to megacolon, ileus, sepsis, or even death [2]. National epidemiological investigations have demonstrated significant increases in CDI incidence in the United States (U.S.) in recent years. A recent study found that the rate of CDI in U.S. community hospitals increased two-fold between 2001 and 2010 [3].

Cancer has been previously found to be associated with the development of CDI. One study found that the rate of hospital-onset CDI was twice as high among cancer patients as compared to all other inpatients [4]. This association is likely due to a number of factors. First, cancer patients might have a greater degree of exposure to *C. difficile* due to frequent or prolonged hospitalizations. Second, immunosuppression from the disease or drug therapy could predispose cancer patients to develop clinical infection, rather than colonization. Furthermore, prior studies have shown that immunosuppressed patients who develop CDI are at higher risk for poor clinical outcomes [5, 6]. Lastly, cancer patients are frequently exposed to medications and other factors that can alter the gut microbiota, including certain chemotherapeutic agents, antibiotics, gastric acid suppressing medications, and manipulation of the gastrointestinal tract (e.g., enteral feedings). As gut microbiota play a role in preventing *C. difficile* colonization and virulence, these alterations can lead to CDI [7].

Despite this association, the national rate of CDI among cancer patients is unknown. Furthermore, it is unclear how CDI affects clinical outcomes in cancer. Therefore, the objectives of this study were to describe longitudinal trends in CDI incidence among hospitalized cancer patients in the U.S., describe trends in mortality and inpatient length of stay (LOS) in cancer patients with CDI, and compare outcomes in cancer patients with and without CDI.

## Methods

### Data source

The data source for this study was the U.S. National Hospital Discharge Surveys (NHDS) from 2001 to 2010. These surveys contain a national probability sample representative of the U.S. population discharged from community hospitals annually. Staff from the participating hospital, U.S. Census Bureau, or National Center for Health Statistics collected NHDS survey data manually or automatically, including *International Classification of Diseases, Ninth Revision, Clinical Modification* (ICD-9-CM) codes for diagnoses and procedures. Sampling and collection methods allow the user to apply data weights to hospital discharge data to derive national estimates, as previously described [3, 8]. The datasets are publically available through the U.S. Centers for Disease Control and Prevention and contain no patient identifying information; therefore, this study was considered non-human subjects research by the Institutional Review Board of the University of Texas Health Science Center at San Antonio.

### Study design

This was a retrospective analysis of patients ≥18 years old with a principal or secondary ICD-9-CM discharge diagnosis of cancer (140–165.X, 170–176.X, 179–189.X, 190–209.XX). Blood cancers were identified by codes 200–208.XX, while all other patients were considered to have solid cancers. Patients with a principal or secondary diagnosis code for CDI (ICD-9-CM code 008.45) were identified, and formed subgroups for analysis. Principal CDI refers to patients with an ICD-9-CM code in the first position, which generally denotes the primary reason for hospitalization. Secondary CDI refers to patients having an ICD-9-CM code in any other position, and represents a contributory diagnosis not primarily responsible for hospitalization. Diagnostic procedures vary by facility, and are not indicated in NHDS survey data.

Patient baseline characteristics included sex (male or female), race (categorized as white, black or other), geographic region as defined by the U.S. Census Bureau, hospital size (6–99 beds, 100–199 beds, 200–299 beds, 300–499 beds, or ≥500 beds), principal payment source (private, Medicare, Medicaid, self-pay, or other), and admission type (emergency, urgent, elective), as previously described [3, 8]. Mortality was defined as all-cause, in-hospital mortality and was derived from the “discharge status” item of the NHDS. Length of stay was calculated using the “days of care” item of the NHDS.

### Data and statistical analyses

Annual and total CDI incidence rates were determined by dividing CDI discharges by cancer discharges. Rates were presented as principal, secondary, and overall CDI discharges per 1000 total adult cancer discharges. We also characterized CDI incidence for blood and solid cancers. Patient characteristics were described as median (interquartile range) for continuous quantitative variables and counts (percentages) for nominal categorical variables.

Baseline characteristics were compared between adult cancer patients with or without a CDI diagnosis using bivariable analysis (Wilcoxon rank sum test for continuous variables and chi-square test for categorical variables). An alpha level < 0.0001 was used to determine statistical significance due to the large sample size. Independent predictors of mortality and LOS were identified using multivariable logistic and linear regression, respectively, and the following covariates: age, gender, race, marital status, season, geographic region, and number of hospital beds. Odds ratios (OR) and 95% confidence intervals (CI) were reported for the model.

## Results

### Baseline characteristics

Table 1 provides an overview of the study population. These data represent approximately 30,244,426 cancer discharges from U.S. hospitals from 2001 to 2010. CDI was present in 260,219 (0.9%) cancer patients. Of these, principal and secondary CDI occurred in 64,933 (25%) and 195,286 (75%) patients, respectively.

**Table 1:** Patient demographics (*n* = 30,244,426)

Demographic	No CDI (*n* = 29,984,207)	CDI (*n* = 260,219)	*P* value^a^
Age (y), median (IQR)	69 (57–78)	71 (61–78)	< 0.0001
Sex, %			< 0.0001
Male	50.2	51.3	
Female	49.8	48.7	
Race, %			< 0.0001
White	81.5	87.4	
Black	14.4	7.6	
Other	4.1	5.0	
Geographic Region, %			< 0.0001
Midwest	26.1	24.2	
Northeast	22.5	26.4	
South	37.0	32.0	
West	14.4	17.4	
Hospital Size, %			< 0.0001
6–99	18.1	11.1	
100–199	22.6	21.2	
200–299	20.0	25.3	
300–499	23.4	24.9	
Over 500	15.8	17.5	
Principal payment source, %			< 0.0001
Medicare	54.0	60.6	
Medicaid	8.0	5.6	
Private	32.5	31.6	
Self-pay	2.6	0.7	
Other	3.0	1.4	
Admission type, %			< 0.0001
Emergency	43.4	55.0	
Urgent	22.1	28.0	
Elective	34.5	17.0	

*IQR* interquartile range, *CDI Clostridium difficile* infection

^a^P values represent comparisons between CDI and no CDI diagnosis groups

Cancer patients with and without CDI differed significantly in age, sex, race, geographic region, hospital size, principal payment source, and admission type. Patients with CDI were more likely to be older (median age 71 vs. 69 years; *p* < 0.0001), male (51.3% vs. 50.2%; *p* < 0.0001), residents of the Northeast region (26.4% vs. 22.5%; *p* < 0.0001), and Medicare users (60.6% vs. 54.0%; *p* < 0.0001). CDI patients’ admission type was also more often emergency or urgent (55.0% vs. 43.4% and 28.0% vs. 22.1%, respectively).

### CDI incidence

**Fig. 1:**
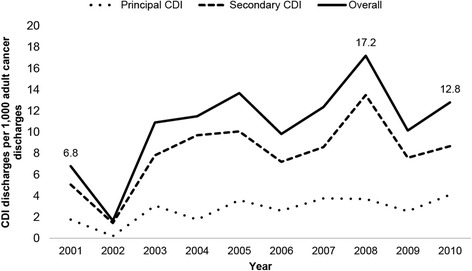
CDI incidence among U.S. hospitalized adults diagnosed with cancer, 2001–2010

From 2001 to 2010, the overall CDI incidence was 8.6 discharges per 1000 adult cancer discharges. The incidence increased from 6.8 per 1000 cancer discharges in 2001 to 12.8 in 2010 (Fig. 1). Incidence peaked in 2008 (17.2 per 1000 cancer discharges). Principal CDI also increased during the study period, rising from 1.8 per 1000 cancer discharges in 2001 to 4.1 in 2010. Secondary CDI incidence increased from 5.1 per 1000 cancer discharges in 2001 to 8.7 in 2010. The incidence of CDI among blood cancers patients (17.3 per 1000 blood cancer discharges) was higher than that of solid cancers (6.8 per 1000 solid cancer discharges).

### Mortality

**Fig. 2:**
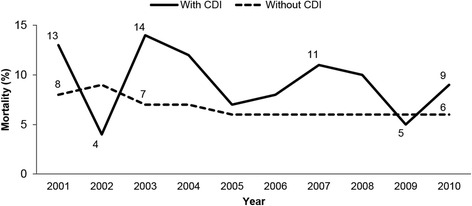
Mortality among U.S. hospitalized adults diagnosed with cancer with and without CDI, 2001–2010

Death occurred in approximately 7.4% of cancer patients during the study period, representing 2,228,061 adult deaths. The mortality rate was significantly higher for cancer patients with CDI compared to without CDI (9.3% vs. 7.4%, *p* < 0.0001). This trend was persisted over the study period (Fig. 2). Mortality for those with CDI was only lower than those without in 2002 and 2009. Mortality among cancer patients without CDI was relatively stable across the study period, dropping from 8% in 2001 to 6% in 2005 and remaining at this level until 2010. In the multivariable model, CDI independently predicted in-hospital mortality (OR 1.03; 95% CI 1.01–1.04), albeit to a small degree.

### Hospital LOS

**Fig. 3:**
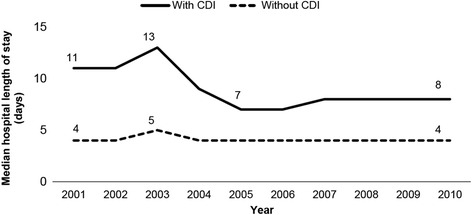
Median hospital length of stay among U.S. hospitalized adults diagnosed with cancer with and without CDI, 2001–2010

Overall median (interquartile range) LOS for cancer patients was 4 (2–8) days. The median LOS was significantly longer for cancer patients with CDI compared to without CDI (9 days vs. 4 days, *p* < 0.0001). Median LOS for cancer patients with CDI dropped slightly from 11 days in 2001 to 8 days in 2010, peaking at 13 days in 2003 (Fig. 3). Median LOS for cancer patients without CDI remained comparatively lower and relatively consistent throughout the study, with 4 days in all study years except 2003 (5 days). Among CDI patients, median hospital LOS was longer for blood cancers compared to solid cancers. CDI independently predicted increased hospital LOS (OR 4.16; 95% CI 4.12–4.20).

## Discussion

This study documents the national epidemiology of CDI among cancer patients discharged from U.S. community hospitals over a 10-year period. We found that CDI incidence increased among cancer patients from 2001 to 2010. Furthermore, cancer patients with CDI are at greater risk for mortality and a longer hospital stay.

The rates of CDI among cancer patients found here are relatively large compared to the general population of U.S. hospitalized adults. In a study using the complete NHDS survey data sample, Reveles et al. [9] found that CDI incidence estimates in the general population of U.S. hospitalized adults grew from 4.5 to 8.2 CDI discharges per 1000 total discharges between 2001 and 2010. In parallel with the cancer population, overall CDI incidence was found to peak in 2008. In an analysis of data from the Healthcare Cost and Utilization Project, Lucado et al. [10] found that between 2000 and 2008, the U.S. saw a 2.5-fold increase in the number of hospitalizations with any CDI discharge diagnosis. The number of hospital stays stabilized between 2008 and 2009, however. In Canada, reports of CDI epidemics were found to occur around the same period [11–14], with mortality increasing drastically between 1997 and 2005. And although limited information on the increased incidence of CDI is available across Europe, a network of laboratories in 34 European countries estimated a CDI incidence of 4.1 per 10,000 patient-days per hospital (range 0.0–36.3) in 2008 [15, 16].

The rise of CDI has been partially credited to the spread of the hypervirulent strain of *C. difficile* categorized as North American pulsed-field Type 1, restriction enzyme analysis type BI, and PCR ribotype 027 (NAP1/BI/027). By 2008, CDIs due to the NAP1/BI/027 strain were reported in 40 U.S. states and across Canada, becoming endemic in some North American healthcare settings [17]. In a survey evaluating the spread of ribotype 027 in Europe, this *C. difficile* strain had been found in 16 European countries by 2008 [18]. This same year, Bauer et al. estimated a 5% prevalence of ribotype 027 across 34 European countries [16]. More recently, the emergence of the ribotype 078 has been associated with disease in younger patients more frequently prescribed fluoroquinolones and with community-associated or indeterminate CDI, as compared to ribotype 027 patients in the Netherlands [19].

Few prior studies have evaluated the rate of CDI in cancer. Kamboj et al. [4] conducted a survey of 11 U.S. cancer centers, aiming to determine the rate of hospital-onset CDI (HO-CDI) in hematopoietic stem cell transplant (HSCT) and cancer patients. Centers using polymerase chain reaction (PCR) as a detection method were found to have a higher median HO-CDI rate compared to those using enzyme immunoassay (1.72 vs. 0.9 per 1000 patient days, respectively), although both rates were higher than those reported for U.S. patients overall. A retrospective review of leukemia patients revealed that CDI occurred in 7% of all cycles of myelosuppressive chemotherapy. Lastly, an analysis of 134 patients found that CDI occurred in 18% of patients with acute myeloid leukemia and in 9% of all treatment courses [20, 21].

Prior studies have demonstrated less favorable health outcomes among cancer patients who develop CDI compared to those who do not. In a retrospective analysis of 186 U.S. hospitals, Campbell et al. [22] found that high-risk patients suffering from HO-CDI, including those with cancer, have significantly longer LOS compared to non-CDI controls. Similarly, a retrospective cohort study found that CDI inpatients receiving chemotherapy for hematologic malignancies had greater mean length of stay compared to similar patients without malignancies. This increase in stay was largely attributed to cancer-related care, as the cancer patients often required neutropenia management, total parenteral nutrition, or pain control [23]. In a study of 5594 adult patients receiving cancer treatment with CDI, CDI-related mortality was 19.7% [24]. This is greater than the 9.1–16.3% mortality reported by others, perhaps due to differences in duration of neutropenia or dissimilarities in study populations [25, 26]. Neutropenia was found to independently predict CDI-related mortality in these patients [24].

Cancer patients maintain a particularly high risk for CDI. Given their frequent or prolonged hospitalizations, patients may have a greater degree of exposure to *C. difficile*. Duration of hospital stay has been previously linked to CDI, as well as recurrent CDI [27, 28]. Prince et al. [29] reported that 32% of cancer patients undergoing chemotherapy had at least one hospitalization, and cancer inpatients have been found to have longer median LOS compared to non-cancer patients. In a retrospective study of inpatients, 36% of cancer patients were found to have LOS > 7 days compared to 26% of non-cancer patients [30]. Schuller et al. [31] found that over the course of a year, 13% of patients on a pediatric oncology ward developed CDI. Analysis illustrated that duration of hospital stay was a primary determinant of infection, given patients’ increased likelihood of intensive neutropenia treatment or long-term antibiotic exposure. In 2009, NHDS data estimated that the average LOS for an adult primary cancer diagnosis was 1.6 days longer than a non-cancer diagnosis, with secondary malignancies, lung cancer, and prostate cancer leading in number of inpatient discharges [32].

CDI rates varied based upon cancer type. CDI was found to be over 2.5 times more common among patients with blood cancers compared to those with solid cancers. This disparity may be due to disproportionate CDI risk factors among blood cancer patients. First, patients with blood cancers might receive antibiotics at a higher rate due to a higher incidence of neutropenic fever resulting from cytotoxic chemotherapy and direct effects on host immunity [33]. Furthermore, patients with blood cancers tend to have a longer length of stay during hospitalizations compared to solid tumor patients [32]. Lastly, blood cancers have the therapeutic option of HSCT. When comparing HSCT recipients versus other cancer patients, Chopra et al. [5] reported HSCT recipients to have 1.4 times higher CDI rates. It is hypothesized these differences are due to chemotherapy regimens and antibiotic use leading up to transplantation, in addition to prolonged hospital stay [6, 34–36]. The distinction between blood cancer versus solid cancer is of importance in CDI prevention and treatment. Due to the increased risk associated with hematologic malignancies, more diligent antimicrobial stewardship may be warranted along with potentially more aggressive CDI treatment.

Immunosuppression from host immunosenescence, the disease, or drug therapy could predispose cancer patients to clinical infection, rather than colonization, as the patient might not be able to mount as strong of a host response. Older age [16, 27], severe underlying disease [16, 27], and immunosuppressive therapy [37, 38] have all previously been associated with CDI. Furthermore, prior studies have shown that immunosuppressed patients who develop CDI are at higher risk for poor clinical outcomes [5, 6].

Lastly, cancer patients are frequently exposed to medications and other factors that can alter the gut microbiota or alter the host response. The following classes of medications or therapies are used frequently among cancer patients and have been previously associated with CDI: antibiotics [16, 27, 37–39], certain chemotherapeutic agents [40], gastric acid suppressing medications [27], and manipulation of the gastrointestinal tract (e.g., enteral feedings, enemas, stimulants) [41].

Knowledge of the burden of CDI among cancer patients is important for several reasons. First, cancer patients can be more readily identified as a high-risk population in whom antimicrobial stewardship and other infection control processes should be targeted. Furthermore, clinicians might choose CDI therapy differently for cancer patients as compared to non-cancer patients. For example, clinicians might choose a more aggressive or costly therapy in cancer patients to improve clinical outcomes. A prior randomized controlled trial found that, among cancer patients, fidaxomicin use resulted in higher clinical cure rates and fewer recurrences as compared to those treated with vancomycin [42].

This study has limitations, predominately due to its retrospective design. First, use of ICD-9-CM codes to identify CDI and cancer diagnoses could result in misclassification bias, as these diagnoses could not be confirmed. However, a prior study noted relatively high sensitivity (78%) and specificity (99.7%) of the CDI ICD-9-CM code compared to microbiological data [43]. Additionally, data related to specific CDI diagnostic procedures were unavailable and could have affected incidence rates, as more sensitive detection methods (e.g., PCR) have been used more commonly in recent years. Lack of these diagnostic tests and other CDI-specific information precluded the analysis of specific *C. difficile* strains, presence of CDI on admission, and stratification by initial and recurrent CDI episodes. Factors that could have influenced CDI outcomes, but were unavailable to control for in analyses included: medications, other health care exposures, and severity of CDI or cancer. In the case of disease severity, patients with cancer or severe illness may suffer prolonged hospitalization or require additional medications, perhaps leading to increased mortality and LOS overall. Lastly, the NHDS include only community hospitals; therefore, our results might not be generalizable to federal or long-term care hospitals or outpatient facilities.

## Conclusions

CDI incidence increased dramatically among adult cancer patients discharged from U.S. community hospitals between 2001 and 2010. Furthermore, CDI significantly increased the risk for mortality and prolonged hospital stays among cancer patients.

## Abbreviations

CDI: *Clostridium difficile* infection
CI: Confidence interval
HO-CDI: Hospital-onset *Clostridium difficile* infection
HSCT: Hematopoietic stem cell transplant
ICD-9-CM: International classification of diseases, 9th revision, clinical modifications
LOS: Length of stay
NAP1/BI/027: North American pulsed-field Type 1, with a restriction enzyme analysis type BI and PCR ribotype 027
NHDS: National hospital discharge survey
OR: Odds ratio
PCR: Polymerase chain reaction
US: United States
